# The Assessment and Management of the Arthritic Knee: An Update

**DOI:** 10.7759/cureus.11582

**Published:** 2020-11-19

**Authors:** Shehzaad Khan, Peter C Logan, Ajay Asokan, Charles Handford, Thomas Moores

**Affiliations:** 1 Trauma and Orthopaedics, Basildon and Thurrock University Hospital, London, GBR; 2 Trauma and Orthopaedics, Walsall Manor Hospital, Birmingham, GBR; 3 Trauma and Orthopaedics, University College London Hospital, London, GBR; 4 Trauma and Orthopaedics, Queen Elizabeth Hospital, Birmingham, GBR

**Keywords:** knee osteoarthritis/ koa, knee arthroplasty, conservative management

## Abstract

Musculoskeletal disorders represent a significant primary care burden. Presentations pertaining to the painful knee are associated with a wide array of differentials; however, among these, osteoarthritis (OA) is the most common one in patients older than 45 years. We have found that a significant number of onward secondary care referrals are misdirected. Therefore, there is a need for a comprehensive assessment and workup to ensure holistic patient care and timely input from specialist services. In this article, we highlight an approach to the management of the arthritic knee.

## Introduction and background

Musculoskeletal disorders constitute a third of general practice (GP)/family medicine consultations. A significant number of GP referrals for specialist orthopaedic review are deemed to be inappropriate or misdirected, with only 58% of those received considered necessary [1]. The most common site for musculoskeletal pathology presenting in primary care is the spine, followed by the knee [1]. Osteoarthritis (OA) is the most common cause of knee pain in patients over the age of 45 years.

In this article, we outline an assessment approach for patients with suspected OA of the knee, discuss the indications for referrals to specialist services, and present up-to-date evidence regarding the topic.

## Review

Diagnosis

The differentials for adults presenting with knee pain are wide and extensive (Table 1). However, among these, OA is the most common one in patients over the age of 45 years, with a clear female preponderance [2]. The National Institute for Health and Care Excellence (NICE) has published guidelines on the assessment and management of OA, stating that OA can be diagnosed clinically if a person is over the age of 45 years, has activity-related joint pain, and has either a) no morning joint stiffness, or b) morning stiffness lasting no longer than 30 minutes [2].

**Table 1:** A list of differential diagnoses to consider for a painful knee in an adult

Traumatic	Atraumatic
Sprain	Osteoarthritis
Patella tendonitis	Gout/pseudogout
Bone bruising	Inflammatory arthritis
Ligament rupture	Bursitis
Meniscal tear	Septic arthritis
Osteochondral defect	Avascular necrosis
Patella dislocation	Osteochondritis dissecans
Fracture	

Radiographs are the gold standard for the first-line investigation of knee pain. The following views should be included: weight-bearing anterior-posterior (AP), lateral, and merchant views. When combined with a physical examination, the sensitivity and specificity of diagnosing OA in the knee are 91% and 86%, respectively [3].

MRI should not be requested routinely, except in cases of diagnostic uncertainty, or when there is a high clinical suspicion of a possible alternative diagnosis, e.g., osteochondritis dissecans or avascular necrosis. MRI is particularly unhelpful in the elderly population with suspected OA, where incidental pathological meniscal findings are very common but not clinically significant [4,5].

Management

**Figure 1:**
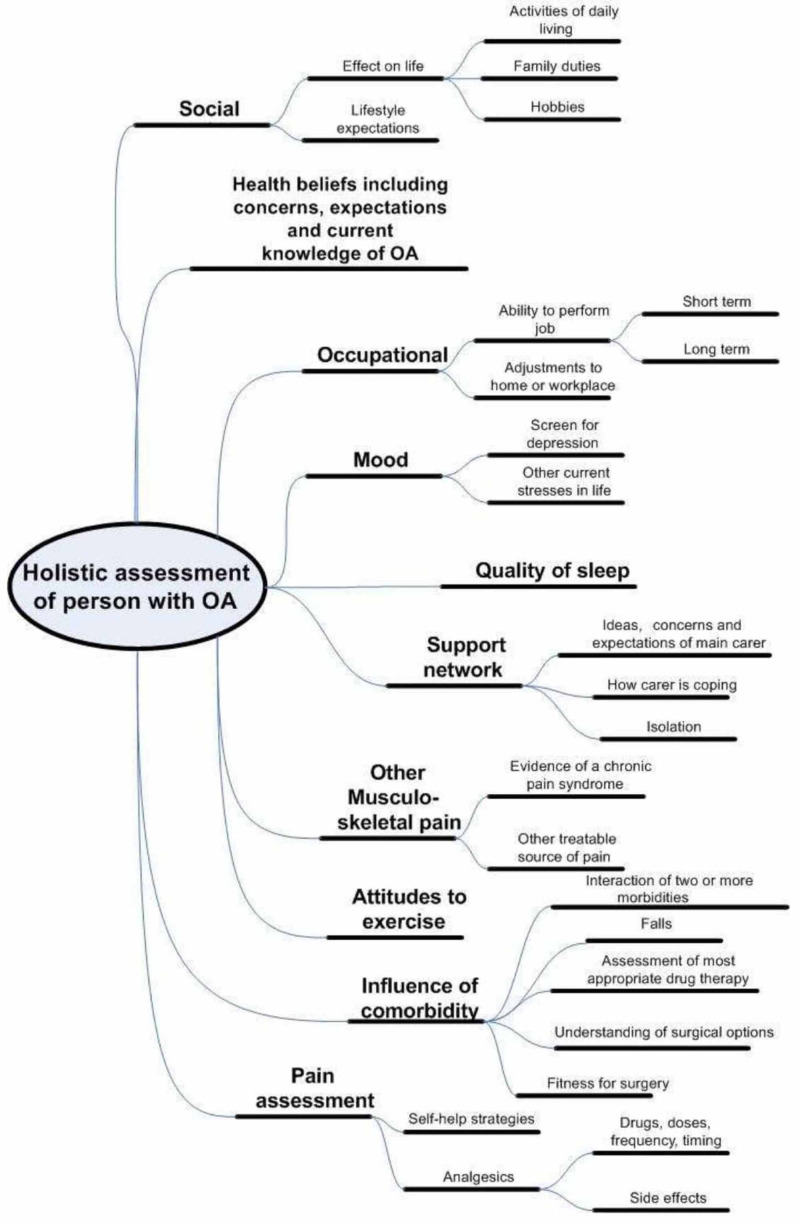
NICE guidelines on the holistic assessment of a patient with OA NICE: the National Institute for Health and Care Excellence; OA: osteoarthritis

NICE proposes a holistic approach to the management of OA (Figure 1).

Non-surgical options that should be initiated in the primary care setting

Weight loss: as trivial as it sounds, this should be one of the first-line treatments offered for symptomatic OA. A reduction in weight decreases the reaction forces across the joint, thereby decreasing pain. Weight loss should be offered before starting weight-bearing exercise to preserve joint integrity. Body mass index (BMI) of the patients must be clearly documented in any onward referrals, as some local organisations (Care Commissioning Groups) will have a BMI cut-off for certain procedures; e.g., no joint replacements with a BMI of >35 kg/m^2^. This is due to the increased risk of infection associated with excess adiposity.

Physical rehabilitation and exercise: in addition to the benefits accorded by weight loss, exercise and thigh-strengthening have also been shown to confer further cumulative benefits. There is level 1 evidence to support improved pain and function after 12 dedicated sessions. The Osteoarthritis Research Society International (OARSI) has endorsed the use of land-based exercise programs as a core treatment for knee OA [6]. Aquatic options should be suggested for patients with serious mobility and functional limitations. This has been quoted as a 'level 1B' therapy in knee OA, with >75% in favour of its use based on a systematic review and analysis [6].

Knee braces and walking aids: offloading knee braces have been shown to be especially useful in unicompartmental OA. However, insoles and wedges have had no significant effects on symptoms.

Pharmacological options: non-steroidal anti-inflammatory drugs (NSAIDs), highly selective cyclooxygenase-2 (COX-2) inhibitors, and tramadol have shown the best evidence for better efficacy and symptom control, as advised by the American Academy of Orthopaedic Surgeons (AAOS). This view has been further supported by the OARSI guidelines, which has found the use of topical NSAID therapy to be a level 1A therapy [6]. These can be advocated in the absence of contraindications, e.g., use of NSAIDs in peptic ulcer disease, and in such circumstances, the concurrent use of a gastro-protective proton pump inhibitor (PPI) can be added.

Alternative treatment methods: options such as glucosamine, chondroitin, viscoelastic joint injections, and acupuncture are controversial and AAOS has provided strong evidence advocating against their use.

Intra-articular corticosteroids: corticosteroid therapy has been shown to provide good short-term relief by directly reducing joint inflammation, without significant systemic side effects. While the risks include tendon rupture, articular cartilage degeneration, and septic arthritis, the use of steroid injections has become increasingly popular as more GPs are trained in its use. Up to 96% of patients have reported satisfaction with corticosteroid injections and a resultant reduction in pain scores. Two key points to note are the concentration of the local anesthetic being used and the timing of the injection:

A) a high dose of local anesthetic is associated with increased chondrolysis and we, therefore, recommend using a diluted dose, e.g., 0.25% levobupivacaine.

B) higher rates of post-total knee arthroplasty infections have been noted in patients who had received an intra-articular corticosteroid injection within three to six months of their operation [7]. Roughly 6-9% of patients receive an intra-articular injection prior to the referral [2]. Therefore, any patient who seeks an orthopaedic opinion regarding surgical interventions should refrain from receiving an intra-articular injection.

Surgical Options

Joint preservation: mechanical symptoms such as ‘locking’ or ‘catching’ have traditionally qualified for arthroscopic procedures, e.g., arthroscopic meniscal debridement. However, evidence has shown that performing such procedures in OA knees results in little to no improvement in symptoms and function when compared to placebo or conservative options [8]. We recommend that patients in this category be referred for physiotherapy instead.

Joint arthroplasty: currently, indications that are considered to refer a patient for arthroplasty are as follows: severe and debilitating pain, especially at night; exhaustion of all conservative measures; functional impairment to the quality of life; radiographic evidence of OA. BMI also plays a role in local organisations' decision-making, with many declining to operate on patients with a BMI greater than 40 kg/m^2^ due to their complication profile, except in individually requested circumstances [9]. Oxford Knee Scores should be measured for all patients to benchmark their symptoms pre- and post-intervention. However, they should not be used to determine a patient’s suitability for surgery [10].

## Conclusions

Based on our findings, the key take-aways are as follows: OA of the knee is a common diagnosis made in primary care. Many of these patients can be managed very comfortably in the community. However, using the above-mentioned criteria and in line with up-to-date evidence, we can highlight the cases of more urgent patients who may require surgical intervention and thereby reduce the referral rates. This would result in shorter waiting lists for outpatient appointments and a better patient journey and outcomes.
